# Correction: Entanglement witnesses and separability criteria based on generalized equiangular tight frames

**DOI:** 10.1038/s41598-026-38434-6

**Published:** 2026-02-05

**Authors:** Katarzyna Siudzińska

**Affiliations:** Institute of Physics, Faculty of Physics, Astronomy and Informatics, Nicolaus Copernicus University in Toruń, ul. Grudziądzka 5, 87–100 Toruń, Poland

Correction to:* Scientific Reports* 10.1038/s41598-025-13403-7, published online 14 August 2025

The original version of this Article contained errors.

Equation 79 was incorrectly given as:

$$\begin{array}{c}{\Vert \mathcal{R}\Vert }_{\mathrm{tr}}=\sqrt{{\widetilde{\mathcal{C}}}^{A}-{\mathcal{C}}^{A}\left({\rho }^{A}\right)}\sqrt{{\widetilde{\mathcal{C}}}^{B}-{\mathcal{C}}^{B}\left({\rho }^{B}\right)}\end{array}$$

The correct Equation 79 appears below.

$$\begin{array}{c}{\Vert \mathcal{R}\Vert }_{\mathrm{tr}}\le \sqrt{{\widetilde{\mathcal{C}}}^{A}-{\mathcal{C}}^{A}\left({\rho }^{A}\right)}\sqrt{{\widetilde{\mathcal{C}}}^{B}-{\mathcal{C}}^{B}\left({\rho }^{B}\right)}\end{array}$$

The original Article has been corrected.

